# Best Practice in Systematic Reviews: The Importance of Protocols and Registration

**DOI:** 10.1371/journal.pmed.1001009

**Published:** 2011-02-22

**Authors:** The PLoS Medicine Editors

It is now just over six years [1] since many medical journals began requiring that trials be registered before considering the trial report for publication. Such a policy was set up explicitly to reduce what was considered to be widespread bias in favor of publication of “positive” trials and to ensure that all clinical trials be made public prior to participant enrollment. Given the importance of clinical trials for estimating the efficacy and safety of interventions, and their role in approval of new drugs and devices, such a policy seemed uncontroversial. Although it is known that some trials still go unregistered, there are strong incentives (such as journal publication) and, in some countries legally enforceable mandates, for authors to register these studies before enrolling patients. The existence and widespread uptake of trial registration helps researchers, patients, and funders understand how many trials are being undertaken and which interventions are being evaluated. It also allows studies to be traced from inception through to completion and publication [2].

However, well-conducted systematic reviews—overviews of health care interventions that use a predefined, explicit methodology to find and synthesize all the relevant evidence—are generally considered higher-caliber evidence than are individual trials in decision-making for clinical practice and health policy. The superiority given to such reviews derives from key aspects inherent to the process of carrying out a systematic review. This study type, if done properly, allows the review to come closer to estimating the true effect of an intervention than any single study can, for two main reasons. First, such reviews collect and synthesize all relevant studies; second, reviews appraise each included study for risk of bias.

However, there is increasing evidence of the existence of publication bias for systematic reviews. A recent survey [3] indicates that nonpublication of completed studies may be as much of a problem for systematic reviews as it is for trials. Other analyses [4],[5] point to the existence of discrepancies between systematic review protocols and the published report, with one study [5] showing that the outcomes included in published systematic reviews may be biased toward “positive” findings. It is crucially important, therefore, that if the evidence from these studies is to be incorporated into clinical practice, the review is as rigorous and as fully reported as possible. For example, it should be obvious to readers whether there was a prespecified protocol for the review, that deviations are noted, and whether outcomes from the review are reported according to the original study plan. Increased clarity surrounding systematic review conduct and reporting would be possible if the protocols for systematic reviews, just like those for trials, were registered [6],[7].

Systematic reviews conducted under the auspices of the Cochrane Collaboration are registered early, at the protocol development stage. This registration helps minimize bias in the conduct and reporting of the review, reduce duplication of effort between groups, and keep systematic reviews updated. However, until now no overarching registry open to all researchers, worldwide, has existed for recording the existence and development of systematic reviews from inception through to completion.

This month, the Centre for Reviews and Dissemination (University of York, UK), supported by the UK National Institutes of Health Research and in collaboration with an international advisory group, announces PROSPERO, its international Prospective Register of Ongoing Systematic Reviews. Following months of public consultation, with many hundreds of respondents from 34 countries providing input on the proposed registration process and minimum dataset, PROSPERO is now open for business [8]. Registration is free, is available to anyone around the world, and generates a unique identifying number for each registered systematic review, which can (and should) be reported in any publications that arise from the study. Investigators should use the registry to record the existence of the protocol for a planned or ongoing systematic review of health care interventions even before screening studies for inclusion in the systematic review. A minimum dataset specifies the key items that are required for a systematic review to be meaningfully registered. Key data items include a statement of the research question, patients and population, study intervention(s) and outcomes; criteria for inclusion and exclusion of studies in the systematic review; outline of search strategy; and methods to assess risk of bias and for analysis of studies included in the systematic review.

With a clear system in place for registration of new and ongoing systematic reviews, *PLoS Medicine* announces its support for this initiative. The journal wishes to promote best practice in the conduct and reporting of systematic reviews. Best practice includes registration during the protocol phase in PROSPERO or other appropriate registry, conduct of the review in accordance with a fully developed protocol, and reporting in line with the PRISMA guidelines [9]. *PLoS Medicine* and other PLoS journals will now start asking authors on submission whether registered their systematic review, and if so, to provide us with the registry number, which will be included in the final published article if the study is accepted for publication in the journal. We will also encourage authors to submit copies of their protocols, which will be available for reviewers and editors as part of the review process, and then published as supporting information alongside the full report of the systematic review.

We recognize that it is still early days for registration of systematic reviews. As a result, the *PLoS Medicine* editors are keen to hear from our readers and authors about this new initiative. We recognize that efforts such as this cannot alone eliminate bias in the conduct and reporting of research. We also appreciate that an additional burden is posed to prospective authors; as such we will reassess the PLoS policy on systematic review registration within a year. The research community is still in the process of learning what the publication outcomes are of cohorts of trials registered in the main registries, such as ClinicalTrials.gov and ISRCTN, since these sites were set up and widely supported by medical journals [2]. It will be some time before the uptake and outcomes of systematic review registration are known. We hope, however, that the future success of this initiative will contribute toward increased rigor and transparency of the systematic review literature.
